# Threats to public figures and association with approach, as a proxy for violence: The importance of grievance

**DOI:** 10.3389/fpsyg.2022.998155

**Published:** 2022-10-26

**Authors:** David V. James, Frank R. Farnham, Philip Allen, Ance Martinsone, Charlie Sneader, Andrew Wolfe Murray

**Affiliations:** ^1^Theseus Ltd., Chippenham, United Kingdom; ^2^Fixated Threat Assessment Centre, London, United Kingdom; ^3^Department of Security and Crime Science, University College London, London, United Kingdom

**Keywords:** grievance, violence, fixation, mental health, security, lone-actor grievance-fuelled violence

## Abstract

The adoption of the term grievance-fuelled violence reflects the fact that similarities exist between those committing violent acts in the context of grievance in different settings, so potentially allowing the application of insights gained in the study of one group to be applied to others. Given the low base rate of violence against public figures, studies in the field of violence against those in the public eye have tended to use, as a proxy for violence, attempts by the individuals concerned to achieve unwarranted and unwanted proximity to the subject of their attention, given that approach is a necessary prerequisite for most forms of attack. In such studies, one factor that has frequently been considered is whether the making of threats is associated with a subsequent approach. The results have been varied, with no correlation found in some, a negative correlation in others, and a positive correlation in at least one. Such studies have been retrospective, using case files prepared for other purposes, and samples of cases have been selected according to their victims’ sector of employment – for instance, politicians, celebrities, judiciary, and the corporate world. This study of a sample of 126 threat assessment cases, using a prospective methodology, looks at the associations between the making of threats and subsequent approach from a different angle – that of a standardised and validated classification of underlying motivation. It finds that particular types and forms of threat are significantly associated with subsequent approach in cases that are fuelled by grievance, but not in those with the motivation of seeking a relationship. Furthermore, when a sample with a mixture of motivational categories was examined in the manner of previous studies, such associations with threat were not apparent. These results refine the existing understanding of the significance of threats in public-facing cases. Future research projects in this area might usefully incorporate the consideration of underlying motivation, in particular grievance.

## Introduction

The term grievance-fuelled violence has been used over the last decade to reflect the fact that similarities exist between those committing violent that acts in the context of grievance in settings previously studied separately, such as school shootings, workplace violence, random massacres, and lone-actor terrorism (e.g., Lankford, 2012; McCauley et al., 2013; Capellan, 2015; Clemmow et al., 2022). This is relevant because insights gained in the study of one group may prove to be applicable to others. Indeed, Clemmow et al. (2022) suggest that, rather than developing risk assessment tools for specific contexts of violence, “an overarching framework for guiding threat assessment of LAGFV [*lone-actor grievance-fuelled violence*] may be feasible”. In the field of violence against those in the public eye or in public-facing roles, studies have been similarly “siloed,” with the cases studied according to sector of employment – for instance, celebrities (Dietz and Martell, 1989; Dietz et al., 1991a; Meloy et al., 2008), politicians (Dietz and Martell, 1989; Dietz et al., 1991b,Scalora et al., 2002a,b), royal families (Fixated Research Group, 2006; James et al., 2010; van der Meer et al., 2012), judicial officials (Calhoun, 1998; Eke et al., 2014), and the corporate world (James et al., 2022). The relevance of the concept of grievance-fuelled violence in these areas of study has been described (Pathé et al., 2018; Wilson et al., 2018, 2021; Barry-Walsh et al., 2020, p. 482).

A focus within this literature has been the issue as to whether detectable warning behaviours precede incidents of violence (Meloy et al., 2012, 2021), and one question that has repeatedly arisen is whether the making of threats is associated with subsequent adverse consequences. Some studies have focussed on the cases of physical violence committed against those in the public eye and examined antecedent events for the presence of warning behaviours, including threats (e.g., Fein and Vossekuil, 1999; James et al., 2007, 2008; Hoffman et al., 2011). A general finding is that acts of violence against public figures often involve forms of previous warning behaviour, including the making of threats. This is important as it offers the possibility of preventive interventions. However, such studies offer no information as to the proportion of all communicated threats that are associated with subsequent violence. A problem with trying to examine the associations of threats prospectively is the very low base rate of violence. Given this, studies have tended to use, as a proxy for violence, attempts by concerning correspondents at subsequent approach, in other words attempts to achieve unwarranted and unwanted proximity to the subject of their attentions. Approach is considered a proxy for violence because it is a necessary prerequisite for almost all forms of attack.

The method adopted in such studies of threats to public figures is to examine a sample of the writers of inappropriate and concerning letters, comparing those who subsequently approached the public figure with those who did not. Results have generally shown that, although many individuals who approach have made threats, there is no significant association between making threats and approaching in some studies: In a few studies, a positive association was indeed identified; yet in others, there was a significant inverse correlation between the two. To an extent, the differences in results may be influenced by the sector of employment of the public figures forming the basis of each study, but even then, the results from research efforts are inconsistent.

Three areas of employment have been subjected to particular study. Dietz et al. (1991a) made an exhaustive study of cases of threatening and otherwise inappropriate letters to Hollywood celebrities, using stratified samples of 107 individuals who pursued encounters with the celebrities and 107 who did not. Of the 214 cases, 49 (23%) made threats of some sort. The definition of threats was broad and not restricted simply to the threats of violence. There were no significant associations, positive or negative, between making threats and subsequent approach. This applied regardless of the type of threat, the consequence threatened, or the number of threats made. By contrast Meloy et al. (2008, 2011) examined an archival, convenience sample of stalkers and harassers, taken from the case files of police, prosecutorial agencies, and the security department of an entertainment corporation, as well as their own personal case archives. They identified 271 cases of “celebrity stalkers,” of whom 18% (48 cases) made threats of physical violence. Threats of physical violence were found to be significantly associated with approach.

A second group of studies concerned politicians. Dietz and Martell looked at threatening and otherwise inappropriate letters to members of Congress (1989; Dietz et al., 1991b), comparing 43 subjects who pursued encounters with the members of Congress with 43 who did not. Threats in some form were made in 50 cases (58%). Threats were significantly associated with the *absence* of subsequent approach. This applied to direct threats, indirect threats, and veiled threats, and also to any form of threat, as well as threats of violence. In addition, the mean number of threats was significantly greater in those who did not subsequently approach. Subsequently, Scalora and colleagues looked for the associations between threats and approach to the members of congress as a part of three broader studies. The first study examined 4,387 electronic case files from the archives of the Threat Assessment Section of the U.S. Capital Police, which concerned threatening or otherwise problematic contacts with members or staff (Scalora et al., 2002a). These cases were then divided into those that approached and those that did not. In all, 986 cases (22.5%) approached. The sample included cases where approach had not been preceded by communication. Subjects “were considered to have utilised threatening language if they described a desire to physically harm or have physical harm occur to the target in either a direct or veiled fashion.” Using this definition, 31.7% of cases involved a direct or veiled threat towards members of the “congressional community.” Approachers were significantly less likely to have made a direct or veiled threat prior to the problematic contact. However, it was noted that 21.5% of approachers had threatened, as had 42% of “violent approaches” (those involving “threat or use of a weapon, attempted, or actual assault”). A second study from the same group (Scalora et al., 2002b) randomly selected 316 of the more recent files from the same source, with a particular focus on pre-approach behaviour. Cases involving threatening language were “over-sampled” in this design to ensure that an adequate proportion of such cases was included. The definition of threatening language was expanded to include, not only threats of physical harm, but also “vague unspecified harm (e.g., potentially to physical, reputational or political well-being).” In total, 104 cases (32.9% of the total sample) approached, and 205 (64.9%) threatened. The making of threats was significantly associated with not making an approach. A third study (Schoeneman et al., 2011) tightened the methodology somewhat in terms of rationale and hypotheses, the emphasis of the study being on thematic content and language characteristics. The authors examined a sample of 326 written contact cases, 49 of whom had engaged in approach behaviour and 277 had not. No significant difference was found between approachers and non-approachers in the use of “threatening language,” for which the operational definition in the coding manual was not included in the published paper.

In a third group of studies, the Fixated Research Group (*FRG*)^1^ conducted extensive investigations into those who engaged in threatening or otherwise inappropriate communications towards the members of the British royal family, or made attempts, successful or otherwise, to approach them (Fixated Research Group, 2006). The definition of threats used was that of Scalora et al. (2002b): in other words, it was broad in scope and not restricted to threats of physical violence. The various studies by the FRG which resulted concerned a structured, random sample of 365 cases taken from 5,685 case files held by the Metropolitan Police Force’s Royalty Protection division. The sampling method ensured roughly equal representation for the different categories of behaviour adopted, these being: pre-approach (i.e., communications); pre-approach and approach; approachers; failed breachers of security cordons; and successful breachers. In comparing those who communicated with those who both communicated and approached, the making of threats was significantly associated with the absence of approach. However, this did not hold for those who made successful or unsuccessful attempts to breach security perimeters for the purpose of achieving proximity. A similar exercise was subsequently undertaken with 107 cases who sent disturbing communications and/or made problematic approaches to members of the Dutch royal family (van der Meer et al., 2012). Here, threats were defined as communications threatening serious physical or sexual assault, kidnapping, or arson. Those who made threatening communications were significantly less likely to approach.

There has been a range of other studies concerning threats to public figures, which have produced valuable findings, but did not directly consider the relationship between threats and approach. For instance, Calhoun (1998) examined more than 3,000 threatening contacts and assaults against federal judicial officials and examined which forms of threat were more likely to be associated with violence. However, concerning communications which did not threaten were not examined. Eke et al. (2014) examined reoffending rates at 2 years in those who had threatened or harassed judicial officials in Ontario. Threateners were significantly more likely to engage in violent reoffending, but the study did not produce results that could be compared with the findings detailed above. Such studies are not considered further here.

The initial reaction to the studies concerning threats and approach that were published in the 1990s was somewhat simplistic, at least amongst non-clinicians, with some authors erroneously concluding that threats were either irrelevant or somehow a protective factor against violence in public figure cases. This sentiment was captured by Meloy (2000, p. 161): “Threats are not that big a deal. That’s right, you read it correctly.” Meloy later set out the nature and history of this widespread misapprehension (Meloy, 2014, pp.244-5) and described the current consensus that threats need to be taken seriously in risk assessment. This has been put clearly by Mullen and colleagues who state the practical position in terms of threats in stalking and harassment: “Threats should be regarded as promises. Like many promises, not all are fulfilled, but nevertheless they should be accepted as a commitment to future action until proved otherwise” (Mullen et al., 2000, p. 218).

Problems of interpretation are not aided by inconsistencies in the results from different studies, which serve to indicate that some parameters may be missing in the consideration of the problem. Dietz and Martell (1989, p. 14: 17), in their major report to the National Institute of Justice, drew up risk-factor scales for approach in celebrity cases. Yet, when they tried to apply these to cases involving the members of Congress, the scales failed to distinguish approach-positive cases from approach-negative. The authors note (Dietz et al., 1991b) that the focus and concerns of those contacting/approaching celebrities differed from those concerned with members of Congress. In the celebrity cases, 56% “idolised or worshipped” the celebrity to whom they wrote, whereas only 2% were preoccupied with “injustice to self.” In the congressional cases, 38% were preoccupied with “justice to self” and only 4% were preoccupied with “love, marriage, romance.” In other words, the pattern of motivations differed between the two samples. The papers discussed above have generally examined motivation in terms of such factors as thematic content, adopted roles, preoccupations, and concerns, using *ad hoc* lists of constructs and categories. Only the royal family studies constructed a standardised typology of motivation and applied it to individual cases. All the studies illustrate a wide range of different pre-occupations and concerns within each sample, though these are not directly comparable between all the studies, tend to be overlapping rather than mutually exclusive, and some of the lists are long. They are not necessarily comprehensive, and it is inevitable that retrospective file trawls produce a proportion of cases with insufficient information to divine motivation. The relevant classifications are set out in Table 1. They key point here is that all the studies involved a rag-bag of motivations, mixed in a way that had no meaning *per se* and was unlikely to be directly reproducible.

**TABLE 1:** Motivational categories adopted in eight studies which examined associations between threats and approach.

Celebrities			Politicians			Royal families	
Dietz et al., 1991a: *N* = 214	Meloy et al., 2008 (*N* = 271)	Dietz et al., 1991b (*N* = 86)	Scalora et al., 2002a (JFS) (*N* = 4,387)	Scalora et al., 2002b (JTA) (*N* = 316)	Schoeneman et al., 2011 (*N* = 326)	James et al., 2010 (*N* = 222)	van der Meer et al., 2012 (*N* = 107)
“Role in which celebrity was cast”	“Motivations”	“Role in which member of Congress was cast”	“Thematic content” (selected)	“Thematic content”	“Content characteristics” (selected)	“Motivational categories”	“Motivations”
- Friend or acquaintance 36% - Spouse, potential spouse, or suitor 27% - Lover, potential lover, or would-be lover 26% - Business associates and collaborators 15% - Religious advisors, prophets, and saviours 15% - Enemies 5% - Persons with special powers 5% - Family members 4% - Rescuers 1%	- Seeking a relationship (affectional, or sexual, or both) *N* = 141 (52%) - Seeking help *N* = 53 (20%) - Only wanted to communicate *N* = 36 (13%) - Insulting *N* = 21 (8%) - Offering help *N* = 16 (6%)	- Enemy, persecutor, or conspirator 42% - Rescuer, benefactor or potential benefactor 23% - Business associate or collaborator 12% - Friend or acquaintance 9% - Lover, potential lover, or would-be lover 6% - Beneficiary of the subject 5% - Spouse, potential spouse, or suitor 2%	- Domestic issues 51.1% - Foreign policy issues 16.7% - Help-seeking behaviour 26.2% - Concerns regarding personal entitlements 15.8% - Obscene /sexualised 16.1% - Racial themes 16.6%	- Policy-oriented (“general complaint regarding government activity, anti-government statements”) 35.4% - Target-oriented (“insulting /degrading language, sexist or sexualised references”) 61.7% - Personal oriented (“exclusively relating to the subject, personal help-seeking request, specific entitlement issue”) 23.1%	- Help-seeking requests 85 (26%) - Entitlement claims 28 (8.6%) - Reference to financial difficulty 46 (14.1%) - Personal themes 106 (32.5%) - Help-offering statements 24 (7.4%) - Love or sexual references 26 (8%) - Religious content 90 (27.6%) - Human rights issues 26 (8%) - Corrupt government claims 114 (35%) - Reference to injustice or rights violated 89 (27%) - Perception of personal danger 35 (10.7%)	- Delusions of royal identity: *n* = 61 (24.5%) - Amity seekers: *n* = 37 (16.7%) - The infatuated: *n* = 23 (10.4%) - Sanctuary and help seekers: *n* = 15 (7%) - The royally persecuted: n = 6 (2.7%) - Counsellors: n = 14 (6.3%) - Querulants: *N* = 13 (5.9%) - Chaotic (mental state too chaotic to discern any singularity of purpose): *N* = 28 (12.6%) - Unknown (insufficient information available on file): *N* = 25 (11.3%)	- Chaotic: n = 30 (28%) - Seeking to bring attention to a perceived problem: n = 26 (24%) - Claiming royal identity: n = 14 (13%) - Seeking help: *n* = 9 (8.4%) - Offering advice: *n* = 6 (5.6%) - Holding royal family responsible for their personal situation: *n* = 6 (5.5%) - Seeking a loving relationship 4 (3.7%) - Saving the country/world 3 (2.8%) - Seeking friendship: 2 (1.9%) - Ending a perceived persecution 2 (1.9%) - Financial gain: *n* = 1 (0.9%) - Unknown (insufficient information available on file): = 4 (3.7%)

In effect, the study of groups selected by the recipients’ sector of employment determines that each sample is a hodge-podge of motivations, even if particular ones predominate in a given sector. This might be of no importance in the study of threat and approach if motivation had no effect on the relationship between the two. Yet, if motivation is important, then to study mixed samples risks defeating the purpose: and there are in fact some clear indications that motivation is a determining factor. Whilst the field may not have been originally conceived of as such, threatening and otherwise inappropriate communications to public figures constitute forms of stalking and harassment: and it is clear from the general stalking literature that motivation is central to the assessment of risk in stalking. The most widely accepted motivational classification, that of Mullen et al. (1999), defines five categories of stalkers, including categories concerning grievance and relationship-seeking. Subsequent studies using this classification have shown that associations of risk in stalking vary between the categories, with significant differences in rates of violence, persistence, and recurrence, and in the prevalence of psychosis and attachment problems (MacKenzie et al., 2008; McEwan et al., 2009a,b, 2017, 2018; James et al., 2010a). In terms of threat, differences have been demonstrated, in that explicit threats were significantly associated with violence in approachers in the rejected category (McEwan et al., 2009b). In addition, it has been illustrated that different forms of risk (e.g., persistence, violence, and escalation) carry different associations in terms of risk factors, and that these vary in turn with motivational group (Mullen et al., 2009b: pp. 226-250). This forms the basis of the Stalking Risk Profile (MacKenzie et al., 2009), a leading structured professional judgement tool for the assessment of risk in stalking, the validity of which has been demonstrated (McEwan et al., 2018). It would seem logical that the association between threats and approach should be investigated in terms of the motivation of the problematic individual, rather than the profession of the victim. As Meloy et al. wrote in 2010: “…The issue for future research should no longer be whether threats are important, but which threats and threateners are associated (or not associated) with which form of adverse event, behaviour or motivational type.” Yet, direct analysis of the association between threats and approach (as a proxy for violence) has yet to be undertaken by motivational type.

Other than the underlying motivation, a further factor which has not been subjected to systematic study concerns the immediate purpose of a threat. In other words, with the underlying motivation as background, what is the immediate purpose of the threat to the threatener? For instance, a threat might be a way of simply venting anger or be intended to cause psychological pain to the recipient. Or it might be a paranoid defensive stance, or a straightforward statement of intent. A simple, standardised classification of immediate purpose has been developed (Warren et al., 2014), but this has not up to now been used in the study of threats in concerning communications to public figures and those in the public eye.

This study was conceived with the specific purpose of examining the relationship between threats and approach in a manner based on motivation, rather than the victim’s source of employment. A particular focus was upon the motive of grievance. In addition, it was intended to avoid some of the methodological problems that are evident in other studies. First, some studies have included cases where there has been approach without previous communication: this confuses the issue. Second, all the principal studies outlined above have problems with sequencing: in other words, they are unable to differentiate (and therefore may have included) cases of communicated threat which were made after approach or were delivered at first approach. This introduces impurity into the samples, such that it cannot safely be concluded whether or not threats are a risk factor, because it is unclear whether any relationship is predictive. There are also divergences in terms of the definition as to what constitutes a threat. The most comprehensive definition has been that of Dietz et al. (1991a,b), who defined a threat as “any offer to do harm, however implausible.” This is broad enough to include, not only threats of physical violence, but also threats of suicide, property damage, financial consequences, reputational damage, and persistence. A similar definition was adopted by Scalora et al. in the second of their studies (Scalora et al., 2002b) and in the British royal family studies (Fixated Research Group, 2006; James et al., 2010). However, the other studies outlined above limited themselves to the consideration of threats of physical violence and excluded other threatened consequences (Scalora et al., 2002a; Meloy et al., 2008; van der Meer et al., 2012), whereas Schoeneman et al. (2011) considered only the rather vague category of “threatening language.” This concentration upon the risk of violence is understandable in terms of the fear of homicide, particularly in jurisdictions where members of the public are able to gain access to firearms. However, other types of threatened action are also serious, such as the causing of reputational or financial damage. Nor is there any reason to suppose that those threatening something other than violence are immune from subsequently engaging in acts of violence. These are arguments for studying threats in the round. Finally, it appears that all studies, apart from an examination of threats in corporate cases (James et al., 2022), have been archival in nature, based upon police or security files prepared for purposes other than research. Given that the cases were historical, the researchers had little opportunity to add information from further sources, other than criminal records or police data and, in the case of Dietz and colleagues’ investigation into celebrity cases (1991a), from details reported in the press.

### Aims

The study aimed to examine the links between threats and approach as a proxy for violence, using a clearly defined sample and adopting standardised categorisations of underlying motivation and of the immediate purpose of threats, which have an established basis in the specialist literature.

In doing so, the aim was to examine four principal hypotheses, derived from an examination of the literature described above:

1) The associations between making threats and approach will differ when the underlying motivation is one of grievance, compared with cases where the motivation is the seeking of a relationship.
2) Associations identified between threats and approach in individual motivational groups will no longer be evident, if all the different motivational groups are considered together as one single group.
3) Between motivational groups, different associations with approach will be found according to the immediate purpose of making threats.
4) The form of harm threatened will be linked with the likelihood of subsequent approach.

To explore hypotheses (1) and (2), particular factors were chosen to examine for positive or negative associations with approach. These were drawn from factors considered in previous studies. Specifically, they comprised:

• Making of threats *per se*.
• Making of different types of threats (i.e., direct, indirect, veiled, and conditional)
• Numbers of threats made, both overall and of each individual threat type.
• Substance of the threats (i.e., the consequences threatened)
• Characteristics of language used.
• Immediacy, escalation, persistence, and plausibility.
• Presence of serious mental disorder.

Overall, the study embraced three broader purposes: to expand understanding of the role of grievance in public figure threats, to help make sense of some of the reasons for conflicting results in previous studies of threats to public figures, and to suggest improvements in methodology for future studies.

## Method

The study used a prospective design, in that data items were gathered on open cases as they progressed, rather than from closed files prepared for other purposes.

### Study sample

The original sample comprised 140 consecutive cases referred to a risk assessment company based on the Fixated Threat Assessment Model (Wilson et al., 2021), in which security/police staff work alongside psychiatric personnel. Each case included in the study involved a full risk assessment and investigation, as opposed to the more numerous referrals in which the company was asked only for an outline opinion or initial handling advice. A purpose-designed dataset was completed as each case progressed and entered into a computerised database in an anonymised form. This included copies of all relevant communications and background information, from which names had been redacted to meet data storage requirements.

The 140 cases had either written concerning communications or made concerning approaches, or both. Cases in which concerning communications had been made subsequent to, or at the time of, first inappropriate approach were then excluded, as were cases which involved approach, but no communication. The cases were then divided according to motivation.

### Definitions

#### Threats

A threat was defined as a statement or indication by someone that they will do something unpleasant or harmful to another or cause something unpleasant to happen to the recipient of the threat or someone close to them. The definition included, but was not restricted to physical violence (i.e., infliction of physical injury), and it encompassed other possible forms of harm:

• Threat of a *psychological* consequence, such as fear, distress, emotional pain, or misery.
• Threat of *sexual* violence (i.e., unwanted sexual activity imposed by physical force).
• Threat to *persist*, that a person’s unwanted attentions will not go away.
• Threat to *escalate* unwanted behaviours, for instance by moving from writing to turning up in person.
• Threat of a *legal* consequence, such as prosecution.
• Threat of a *reputational* consequence, such as adverse publicity or exposure, whether through informing named individuals, employers, news media, or similar.
• Threat of a *financial* consequence, such as losses or ruin.

This definition was presumed to be very similar in scope to that of Dietz and Martell (1989).

#### Types of threat

Threats were classified into the following categories:

• A *direct threat:* a straightforward statement of intent. For instance, “I am going to take you to court”; “I am going to get you sent to gaol”; “I am going to kick the living day-lights out of you”; “I am going to kill you.”
• An *indirect threat* tends to be vague, hinted at, and somewhat unclear. The precise negative consequence and the general plan are unspecified. For instance: “You are going to get what you deserve”; “You are going to be sorry.”
• A *veiled threat* is a coded statement in which no explicit intentions are articulated, but rather are implied. The statement clearly hints at a possible negative consequence, but leaves it to the potential victim to interpret the message and give a definite meaning to the threat. For instance, “You wouldn’t like to see me when I’m angry,” or “It would be a shame if anything happened to you.”
• A *conditional threat* is one that threatens a negative consequence unless the recipient does what the threatener wants, e.g., “Unless you do what I ask, I will ruin you”; “If you don’t settle this matter, your family will suffer.”

The different types of threat used and their number were recorded for each case through studying the totality of communications. Classification was undertaken jointly by four of the authors, together reviewing each case and threat.

#### Approach

An approach was defined as any uninvited attempt, successful or otherwise, to achieve physical propinquity to the person concerned. In total, eight different behaviours, which are not mutually exclusive, were recorded separately and then summated into one variable: turning up at offices/places of work; concerning behaviour at offices/places of work; appearing at home address; attempt at personal approach; physically accosting; following; attack or attempted attack; waiting/loitering for. These behaviours are not considered individually below.

#### Plausibility

Whether a threat was empty or realistic was considered in terms of four different criteria:

• Plausible. The threat is one that would be practicably possible to carry out, e.g., “I will write to your employer.” “I will tell your wife that you are having an affair.”
• Fairly implausible. The threat would be possible, but very difficult to carry out. For instance, “I will shoot you,” in a country with no access to guns. Or “My lawyers will be filing criminal and civil actions,” in someone with a case that no competent lawyer would take on, or where the complainant had no means to pay a lawyer.
• Completely implausible: “I will drop a nuclear bomb on you.”
• Practicable, but toothless: “I will write to the Prime Minister and the newspapers.” Or “I will report you to the police.”

### Categorisations

#### Motivational typology

The motivational typology of harassing and stalking behaviour used in this study is that of Mullen et al. (1999), which is generally accepted as the standard (Pinals, 2007). It defines five categories of stalkers: the Rejected, Intimacy Seekers, the Incompetent Suitor, the Resentful, and the Predatory. The motivational classification is incorporated into the structure of the Stalking Risk Profile (*SRP*: MacKenzie et al., 2009; McEwan et al., 2018), a widely used structured professional judgement tool for the assessment and management of risk in harassment and stalking. The version of the classification used here is the adaptation for public figure cases set out in the *SRP* (pp. 69-71). The definitions of each category are set out in Table 2. This includes an additional three categories (Help Seekers, Attention Seekers and the Chaotic, adapted from Mullen et al., 2009a,b). It excludes the “Predatory” category of the typology by Mullen et al., examples of which are very rare in public figure samples and of which there were none in the current dataset. Whereas the public figure typology excludes the “Rejected” category, it was retained here. The “Resentful” group is to all intents and purposes synonymous with the “aggrieved,” and the latter term will be used from this point on. Cases were allocated to SRP motivational group by joint discussion between three authors, including a co-author of the SRP. In three cases where difficulty was experienced in group allocation, the opinion of the lead author of the SRP was sought.

**TABLE 2:** Definitions of motivational categories extracted from Mullen et al. (1999) and MacKenzie et al. (2009).

Motivational category	Description
*The Resentful (“Aggrieved”)*	The resentful seek to frighten or intimidate the victim to exact revenge for a perceived insult or injury, or to achieve restitution. They include querulants and unusually persistent petitioners righteously indignant at supposed injustice and angrily obsessed with a particular, highly personal cause, not infrequently delusional in its elaboration. These include cases where the individual blames the public figure for their supposed persecution. The individual’s behaviour may be sustained by a satisfying sense of power and control that their behaviour gives them. They almost invariably feel justified in their actions and they often present themselves as a victim fighting back against more powerful aggressors.
*Intimacy seekers*	These include the erotomanic, those with morbid infatuations and with a perceived entitlement to an amicable relationship. They also include those with delusions of kinship towards the prominent individual, except where anger and resentment are predominant at initial contact. Also included here are those who are angry at perceived rejection from an earlier delusional relationship: (the primary phenomenon in such cases is the intimacy-seeking).
*Incompetent suitors*	The socially maladroit (whether through personality, intellectual limitation or mental illness) may make unrealistic and inept attempts to establish a friendship or sexual relationship with a public figure. Such approaches are made in hope, rather than with a sense of entitlement. They are generally insensitive to indications that their attentions are unwanted.
*Help Seekers*	Those who are requesting help from the public figure because of the latter’s position and because they do not know to whom else to turn. Such cases are characteristically hapless, hopeless or helpless, rather than angry. This category excludes those seeking help to further personal causes or quests for justice (who belong in the Resentful category) and also those who believe they have some form of special relationship with the prominent person in question (who should be classified as intimacy seekers).
*Attention Seekers*	Those who wish to make grand public statements or draw attention to themselves as part of a desire for self-aggrandisement, or who hunger for notoriety in order to boost their own feelings of self-worth and importance. Those pursuing idiosyncratic causes of their own are not included here and are likely to belong in the Resentful category.
*Rejected*	Those searching for reconciliation or revenge (or both) for perceived rejection by a former intimate, following the breakdown of a close relationship, almost always sexual in nature.
*The Chaotic*	Those whose mental state is sufficiently confused, generally as a result of psychotic illness, that it is difficult to discern any singularity of purpose.

#### Immediate motives for making threats

The sibilant categorisation of Mullen and colleagues (Warren et al., 2014) was adopted. This divides motives into five separate and mutually exclusive categories:

• *Screaming*: To achieve emotional release, the threat is made to release emotional pain. The act of sending the threat in itself brings relief to the threatener.
• *Shocking*: To cause emotional pain, the threatener rejoices in the idea of the emotional pain that the threats will inflict upon the recipient.
• *Shielding*: To get their defence in first, for instance, paranoid people who believe they are about to be attacked in some manner may threaten as a form of self-defence.
• *Scheming*: To make someone do something, conditional threats to make the recipient behave in a certain way.
• *Signalling*: Declarations of intent, a simple statement of what the person is going to do.

Allocation to categories was undertaken through joint discussion between four authors.

### Statistical analyses

Data on the cases in question were entered into a database and analysed using SPSS-26 (IBM Corp., 2019). The chi-square test was used to examine the associations between categorical variables. Where any minimum cell counts were less than 5, Fisher’s exact test was reported. Odds ratios with 95% confidence intervals are also given for each significant association. For the ease of understanding, odds ratios and 95% confidence intervals of less than 1 are reported as reciprocals. To examine the differences between two samples when the dependent variable was either ordinal or continuous, the Mann–Whitney U test for medians was used. An advantage of the Mann–Whitney U test is that it can be used for relatively small sample sizes.

### Effect sizes

The effect size was calculated for each measure of association. Whereas null hypothesis significance testing indicates how likely it is that a result is due to chance, effect size indicates the magnitude (and therefore importance) of the relationship (Nakagawa and Cuthill, 2007). In contrast to *p*-values, the effect size is independent of sample size, which makes it a useful and separate source of information when sample sizes are small or uneven and Type II errors more likely (Siegel and Castellan, 1988). φ was used as the measure of effect size with chi-square testing, and *r* with Mann–Whitney U (Rosenthal, 1991). In general, when the interpretation of effect sizes cannot be contextualised, it is suggested that a value that reaches 0.1 may be considered a small effect, one that reaches 0.3 a medium effect, and 0.5 a large effect (Cohen, 1988).

### Odds ratios

Odds ratios are used as a further way of describing effect size in associations when the outcome is binary. They represent the probability that an event will occur divided by the probability that it will not occur. Confidence intervals can be provided for odds ratios and ORs are easier to compare than measures such as φ. Other studies in this area have presented odds ratios, and the facility for comparison between studies is desirable. Odds ratios are suitable for retrospective cohort studies (McKenzie and Thomas, 2020), and their inclusion here takes account of it being “best to examine study results presented in several ways to better understand the true meaning of study findings” (Norton et al., 2018, p. 85).

### Considerations of sample size

*A priori* sample size calculations are conspicuous by their absence in the studies of threats and approach, including the present study. This reflects the exploratory and preliminary nature of research exercises in this area, one in which sample size calculations “may be of little value in early exploratory studies where scarce data are available on which to base the calculations” (Jones, Carley & Harrison, p. 455).

### Multiple testing

As the purpose of the study was to explore possible associations, multiple testing was used. In the interests of minimising Type II errors, no corrections to significance values were incorporated to compensate for multiple testing. In consequence, conclusions drawn below from *p*-values larger than 0.01 should be treated with caution, in terms of the possibility of Type I errors (those where an association is erroneously found to be significant).

## Results

### Sample selection

Of the 140 cases in the original sample, fourteen were excluded because approach occurred without prior communication, the communication was handed over at the time of approach, or the communication came after the approach. This gave a sample of 126 cases.

The 126 cases were broken down according to motivational group. In total, 51 cases fell into the resentful/aggrieved category. There were 42 cases which fell into either the intimacy-seeking or incompetent suitor groups. The latter two have important differences, in that the intimacy seekers are more likely to suffer from psychotic illness. However, the shared motivation is the pursuit of an intimate relationship. For this reason, the two were taken together for the purposes of the study and are referred to as “relationship-seeking.”

Preliminary analyses were undertaken to determine whether intimacy seekers and incompetent suitors showed any differences in key areas which might make combining them into one relationship-seeking group inadvisable. Examining the proportions who made threats and the proportions making different categories of threats (direct, indirect, veiled, and conditional), there were no significant differences using the chi-square test, and effect sizes were small, ranging from −0.014 to −0.149. Nor were there any significant differences, employing the Mann–Whitney U test, in overall numbers of threats made or numbers of categories of threats used.

The aggrieved and relationship seekers together accounted for 73.8% of the cases in the sample. The remaining 26.2% (33 cases) were distributed amongst four further motivational groups: attention/publicity seeking 5 (4%); help-seeking 13 (10.3%); chaotic 7 (5.6%); and rejected 5 (4.0%). In total, three cases (2.4%) were difficult to classify. (Two were aggressive Twitter campaigns without any obvious motivation, and one involved an inept attempt at fraud and blackmail). The number in each of these groups was too small to warrant separate study. Accordingly, the study focussed on threatening behaviour in the aggrieved and relationship seekers, the association between threats and approach in these two groups, and for the sake of comparison, some analysis of these factors in the study sample of 126 cases, the sample being undifferentiated in terms of motivation.

Of the resentful cases, 13.7% originated from the talent sector, this denoting a group of actors, authors, television presenters, and other performers. A further 17% were private individuals, essentially those of high net worth or well known because of previous careers in politics. In total, 72.5% were in public-facing roles because of their positions in large banking institutions, well-known companies, or non-governmental organisations (“corporate” cases). The equivalent percentages for the relationship-seeking were as follows: talent sector 78.0%; private individuals 12.2%; and corporate cases 9.8%.

### Comparing basic characteristics of the aggrieved with those of relationship seekers

#### Sex

The sex profiles of the two groups were very similar: Resentful men 40 (80%), relationship-seeking men 33 (78.6%): *χ^2^* = 0.028, p = 0.866, φ = 0.018.

#### Age

The age of the cases was available in 70% of cases (65). The age profiles of the two groups were similar: Resentful: mean = 43.21, SD = 13.05; Relationship-seeking: mean = 39.35, SD = 10.51. Mann–Whitney U = 443.50, Standardised test statistic (*z*) = –1.072, *p* = 0.284, *r* = 0.133.

### Associations between threats and approach

A series of analyses was conducted to examine the associations between the making of threats and approach in the aggrieved, in relationship seekers and in the study sample as a whole, undifferentiated by motivation (“all-motivations” group).

#### Proportions of cases that approached

Of the all-motivations group of 126 cases, 40 (31.7%) approached. Of the 51 aggrieved cases, 14 approached (27.5%). Of the 42 relationship-seeking cases, 20 (47.6%) approached. Aggrieved cases were significantly less likely to approach than relationship-seeking cases (*χ^2^* = 4.039, *p* = 0.044; φ = –0.208; OR [95%CI] 2.404 [1.013–5.682].

#### Associations between type of threat and approach in the mixed motivations sample, and in aggrieved and relationship-seeking groups

In the mixed motivations sample, there were no significant associations between the making of any threats and approach (refer to Table 3, which considers the mixed motivations group, the aggrieved and the relationship seekers separately and sequentially). Nor were there any significant associations between approach and the making of specific types of threat. Indeed, the proportions of cases making different types of threats who subsequently approached are similar to the proportions who did not. In the aggrieved cases, the proportion of threateners who approached is larger for each type of threat than the proportions of threateners who did not approach. The reverse is true for all types of threatening in relationship-seeking cases, in that the proportions of approachers who threatened were smaller than the proportions of threateners who did not approach.

**TABLE 3:** Associations between threats and threat types and approach in the mixed motivations group, the aggrieved and relationship seekers.

Item	Number (proportion) of cases making threats	N (%) of approach: non-approach cases positive for category	*χ^2^* *p*	φ	OR (95% CI)
**1) Associations between making threats and approach in mixed motivations group (*N* = 126)**					
Any threats	98 (77.8%)	30 (75.0%): 68 (79.1%)	0.262 NS	−0.046	-
Direct threats	57 (45.2%)	21 (52.5%): 36 (45.2%)	1.248 NS	0.100	-
Indirect threats	45 (35.7%)	15 (37.5%): 30 (34.9%)	0.081 NS	0.025	-
Veiled threats	50 (39.7%)	16 (40.0%): 34 (39.5%)	0.002 NS	0.004	-
Conditional threats	50 (39.7%)	18 (45.0%): 32 (37.2%)	0.692 NS	0.074	-
**2) Associations between making threats and approach in aggrieved cases*(N* = 51)**					
Any threats	45 (88.2%)	13 (92.9%): 32 (86.5%)	0.397 NS	0.088	-
Direct threats	29 (56.9%)	10 (71.4%): 19 (51.4%)	1.669 NS	0.181	-
Indirect threats	20 (39.2%)	9 (64.3%): 11 (29.7%)	5.088 0.024*	0.316	4.255 (1.159-15.624)
Veiled threats	25 (49.0%)	8 (57.1%): 17 (45.9%)	0.510 NS	0.100	-
Conditional threats	30 (58.8%)	11 (78.6%): 19 (51.4%)	3.107 NS	0.247	-
**3) Associations between making threats and approach in relationship-seeking cases (*N* = 42)**					
Any threats	28 (66.7%)	12 (60%): 16 (72.7%)	0.784 NS	−0.135	-
Direct threats	16 (38.1%)	6 (30%): 10 (45.5%)	1.061 NS	−0.159	-
Indirect threats	13 (31.0%)	3 (15.0%): 10 (45.5%)	4.546 0.033*	−0.329	4.717 (1.067-20.833)
Veiled threats	13 (31.0%)	7 (35.0%): 6 (27.3%)	0.293 NS	0.083	-
Conditional threats	9 (21.4%)	5 (25.0%): 4 (18.2%)	0.289 NS	0.083	-

*Statistically significant.

In aggrieved cases, the making of indirect threats was significantly associated with approach. In relationship-seeking cases, the position is reversed, in that the making of indirect threats is significantly associated with the absence of approach. In the aggrieved cases, the association between conditional threats and approach fell short of significance in the current sample (*p* = 0.078), whilst there was an effect size of 0.247.

#### Association between numbers of threats made and approach

In the aggrieved cases, there was a statistical association between numbers of threats and approach in all categories examined, apart from veiled threats; that is for total threats, direct threats, indirect threats, conditional threats, and number of threat categories used (refer to Table 4). In the relationship seekers, the only significant association was a negative one between approach and numbers of indirect threats made. In the mixed motivations sample, the only statistically significant association between numbers of threats and approach was for direct threats.

**TABLE 4:** Association between numbers of threats and approach.

**Association between numbers of threats and approach**			
	Mixed motivational groups *N* = 126	Aggrieved *N* = 51	Relationship seekers *N* = 42
**Mean ranks Mann Whitney *U, p*, *z, r***			
Number of threats	67.69, 60.03 1472.500, NS −1.123, 0.101	31.75, 22.30 150.500, 0.036* −2.101, 0.300	20.30, 21.67 224.000, NS 0.377, −0.059
Number of direct threats	72.66, 59.24 1353.500, 0.033* −2.126, 0.189	33.57, 23.14 153.000, 0.018* −2.373, 0.332	20.18, 21.79 226.500, NS 0.500, −0.078
Number of indirect threats	66.10, 62.29 1616.000, NS −0.643, 0.057	33.71, 23.08 151.000, 0.008* −2.634, 0.369	18.18, 24.52 286.500, 0.039* 2.060, −0.318
Number of veiled threats	63.99, 62.54 1660.500, NS −0.239, 0.021	28.89, 24.18 204.500, NS, −1.114, 0.158	21.60, 20.43 198.000, NS −0.382, 0.060
Number of conditional threats	68.01, 61.40 1539.500, NS −1.083, 0.097	33.64, 23.11 152.000, 0.018* −2.372, 0.329	21.45, 20.57 201.000, NS −0.325, 0.060
Number of categories of threat used	66.61, 62.05 1595,500, NS −0.672, 0.060	33.36, 23.22 156.000, 0.025* −2.235, 0.313	19.65, 22.29 237.000, NS 0.737, −0.115

*Statistically significant.

#### Associations between Mullen threat categories and approach

A consistent finding with the mixed motivations sample, the aggrieved and the relationship-seeking, was that “shocking” was negatively associated with approach (refer to Table 5). In the aggrieved cases, “scheming” was significantly associated with approach, but no such relationship was found in the mixed motivations sample, nor in the relationship seekers. There were too few cases in the shielding and screaming categories for meaningful analysis.

**TABLE 5:** Associations between Mullen threat categories and approach.

**Associations between Mullen threat categories and approach**			
Mullen threat classification	Mixed motivations group *N* = 126	Aggrieved *N* = 51	Relationship seekers *N* = 42
**Number (%) of approach: non-approach cases positive for category**			
*χ^2^*, *p* φ, OR (95%CI)			
Scheming	19 (47.5%): 33 (38.4%) 0.939, NS 0.086	11 (78.6%): 16 (43.2%) 5.088, 0.024* 0.316 4.813 (1.149-20.165)	5 (25.0%): 3 (13.6%) 0.877, NS 0.145
Signalling	6 (15.0%): 7 (8.1%) 1.389, NS 0.105	1 (7.1%): 1 (2.7%) 0.531, NS 0.102	5 (25%): 6 (27.3%) 0.028, NS −0.026
Shielding	3 (7.5%): 1 (1.2%)	0 (0%): 1 (2.7%)	1 (5%): 0 (0.0%)
Shocking	2 (5.0%): 25 (29.1%) 9.395, 0.002* −0.273 7.813 (1.745-34.483)	1 (7.1%): 14 (37.8%) Fisher’s exact 0.041* −0.301	0 (0.0%): 7 (31.8%) Fisher’s exact 0.009* −0.426 2.333 (1.592-3.421)
Screaming	1 (2.5%): 2 (2.3%)	No cases	1 (5.0%): 0 (0.0%)

*Statistically significant.

#### Associations between consequences threatened and approach

Whereas in the mixed motivations sample, there was no association between approach and threats of physical violence, a different picture emerged when looking at the aggrieved and the relationship seekers (refer to Table 6). In the aggrieved, there was a significant association between threats of physical violence and approach. In the relationship-seeking, there was an inverse relation between threats of physical violence and approach, which did not reach significance in this relatively small sample, although the effect size was –0.286. There were insufficient case numbers for meaningful analysis of disruption, financial damage, or sexual violence.

**TABLE 6:** Associations between consequences threatened and approach.

Item	Mixed motivations group *N* = 126	Aggrieved *N* = 51	Relationship seekers *N* = 42
**Number (%) of approach: non-approach cases making threats of specific category**			
*χ^2^*, *p* φ, OR (95%CI)			
Threats of persistence	14 (35.0%): 19 (22.1%) 2.353, NS 0.137	4 (28.6%): 9 (24.3%) 0.096, NS 0.043	8 (40.0%): 7 (31.8%) 0.305, NS 0.085
Threats of escalation	11 (28.2%): 19 (22.1%) 0.550, NS 0.066	3 (23.1%): 8 (21.6%) 0.012, NS 0.015	7 (35.0%): 7 (31.8%) 0.048, NS 0.034
Threats of reputational damage	20 (50%): 39 (45.3%) 0.237, NS 0.043	12 (85.7%): 22 (59.5%) 3.151, NS 0.249	4 (20.0%): 4 (18.2%) 0.022, NS 0.023
Threats of legal action	8 (20%): 17 (19.8%) 0.001, NS 0.003	5 (35.7%): 13 (35.1%) 0.001, NS 0.005	1 (5.0%): 0 (0.0%) 1.127, NS 0.164
Threats of physical violence	13 (32.5%): 27 (31.4%) 0.015, NS 0.011	9 (64.3%): 11 (29.7%) 5.088, 0.024* 0.316 4.255 (1.159-15.624)	3 (15.0%): 9 (40.9%) 3.446, NS (0.063) –0.286
Threats of suicide	5 (12.5%): 8 (9.3%) 0.302, NS 0.049	3 (21.4%): 5 (13.5%) 0.481, NS 0.097	1 (5.0%): 1 (4.5%) 0.005, NS 0.011
Do all the threats contain the same consequence?	9 (22.5%): 37 (43.0%) 4.961, 0.026* −0.198 2.604 (1.105-6.135)	2 (14.3%): 16 (43.2%) 3.729, NS −0.270	6 (30.0%): 11 (50.0%) 1.739, NS −0.203
**Mean ranks Mann Whitney *U, p, z, r***			
Number of different consequences threatened	27.89, 23.17 230.000, NS −1.155, 0.159	15.75, 12.09, 57.500, NS −1.219, 0.243	6.58, 5.30 11.500, NS 0.652, 0.196

*Statistically significant.

#### Plausibility

There were no significant associations between approach and any of the threat plausibility criteria – neither in the mixed motivations group, the aggrieved, nor the relationship seekers. All effect sizes were below 0.1.

#### Associations between threat characteristics and approach

In total, three of the items examined concerned use of language (refer to Table 7). The use of conditional conjunctions (e.g., if, as long as, provided that, on condition that) was significantly associated with approach in the mixed motivations sample and the aggrieved. In the relationship seekers, there was no significant association in the small sub-sample, but the effect size was 0.230. The use of abusive language was significantly associated with approach in the aggrieved, but not in relationship seekers or the mixed motivations group. The use of emotive language produced no significant associations with approach, although the effect size in the aggrieved was 0.204.

**TABLE 7:** Associations between threat characteristics and approach.

	Mixed motivations group *N* = 126	Aggrieved *N* = 51	Relationship seekers *N* = 42
**Number (%) of approach: non-approach cases making threats with specific characteristics**			
*χ^2^*, *p* φ, OR (95%CI)			
Use of conditional conjunctions	22 (55.0%): 26 (30.2%) 7.102, 0.008* 0.237 2.821 (1.194-3.303)	11 (78.6%): 16 (43.2%) 5.088, 0.024* 0.316 4.813 (1.149-20.165)	8 (40.0%): 4 (19.0%) 2.172, NS 0.230
Use of abusive language in threat	17 (42.5%): 27 (31.4%) 1.481, NS 0.108	11 (45.8%): 13 (35.1%) 7.692, 0.006* 0.388 6.769 (1.597-28.687)	4 (20.0%): 8 (38.1%) 1.620, NS –0.199
Use of emotive, disinhibited or highly charged language in threat,	24 (60%): 38 (44.2%) 2.732, NS 0.147	10 (71.4%): 18 (48.6%) 2.129, NS 0.204	9 (45%): 9 (42.9%) 0.019, NS 0.022
Threats in verbal form (i.e., spoken on telephone or in person)	13 (33.3%): 14 (16.3%) 4.608, 0.032* 0.192 2.571 (1.069-6.187)	8 (61.5%): 10 (27.0%) Fisher’s exact 0.043* 0.315 4.320 (1.140-16.371)	2 (10%): 0 (0%) 2.208, NS 0.232
Threat relates to a clearly defined future action by threatener	24 (60.0%): 41 (47.7%) 1.661, NS 0.115	13 (92.9%): 19 (51.4%) 7.485, 0.006* 0.383 12.316 (1.458-104.017)	7 (35.0%): 9 (42.9%) 0.266, NS –0.081
Any expression of immediacy (deadline, rapid resolution)	8 (20.0%): 20 (23.3%) 0.167, NS −0.036	5 (35.7%): 10 (27.0%) 0.369, NS 0.085	3 (15%): 7 (33.3%) 1.867, NS –0.213
Escalation (increase in intensity, frequency, immediacy of seriousness)	4 (10.0%): 12 (14.0%) 0.385, NS −0.055	2 (14.3%): 5 (71.4%) 0.005, NS 0.010	1 (5.0%): 2 (9.5%) 0.309, NS –0.087
Major mental disorder (psychotic illness, major mood disorder, acute or chronic organic disorder)	14 (46.7%): 20 (29.4%) 2.735, NS 0.167,	7 (53.8%): 6 (18.8%) Fisher’s exact 0.019* 0.351 5.056 (1.239-20.626)	6 (42.9%): 8 (53.3%) 0.030, NS –0.033
**Mean ranks Mann Whitney *U, p*, *z, r***			
Length of intrusive behavior	60.83, 44.50 680.000, 0.009* −2.628, 0.268	31.23, 19.66 101.000, 0.007* −2.691, 0.401	16.67, 11.87 58.000, NS –1.578, 0.304
Number of modes of communication used	65.47, 42.46 541.000, 0.000* −3.783, 0.382	32.42,19.17 85.500, 0.002* 3.137, 0.468	15.17, 13.07 76.000, NS –0.706, 0.136

*Statistically significant.

Threats in verbal form were significantly associated with approach in the mixed motivations sample and the aggrieved, and there was an effect size of 0.232 in the relationship seekers. Threats relating to a clearly defined future action by the threatener were highly significantly associated with the approach in the aggrieved, but not in relationship seekers or the mixed motivations group. In the aggrieved, approach was further associated with the number of different modes of communication used (letters, e-mails, telephone, sms, etc.).

The study also looked at immediacy of the threat (e.g., containing deadlines or other expressions of imminence). It found no significant associations with approach, although the relationship seekers showed a negative effect size of −0.213. No associations were found between an escalation in threatening behaviour (i.e., threats becoming more frequent, intense, or serious in the consequences threatened) and approach in any group. However, persistence in terms of length of intrusive behaviour was significantly associated with approach in the all-motivations group and in the aggrieved, with an effect size of 0.304 found in the relationship seekers.

The question was examined as to whether the use of emotive language might depend upon intent category. Given the strong association between the “shocker” category of immediate intent and absence of approach, the item concerning emotive language was recalculated with all shocker cases excluded. The link between approach and the use of emotive language in the threats was highly significant in the mixed motivations sample: 23 (60.5%): 20 (32.8%), *χ^2^* = 7.333, *p* = 0.007, φ = 0.272, OR 3.143 (1.354–7.295), and in the aggrieved sample 10 (76.9%): 6 (26.1%), *χ^2^* = 8.693, *p* = 0.003, φ = 0.491, OR 9.444 (1.924–12.645), but not in the relationship-seeking sample: 9 (45%): 4 (28.6%), *χ^2^* = 0.941, NS, 0.166.

#### Mental disorder

In aggrieved cases where threats had been made, approach was significantly associated with the presence of major mental disorder (refer to Table 7). No such association was found in the relationship seekers.

## Discussion

This is the first study designed to examine the associations of threats with approach to public figures from the perspective of motivation, approach being selected as a convenient proxy for violence. It concentrated upon the aggrieved, comparing them with relationship seekers, setting them in the context of a study sample which involved a mixed group of motivations. Despite a modest sample size, the study was able to demonstrate that there are particular factors associated with approach in the aggrieved who threaten, and that the significance of these is largely lost, if study samples are analysed without taking account of underlying motivation. Similarly, there are particular factors that apply to relationship seekers, but not the aggrieved, although these are fewer in number.

Before considering how the results relate to the extant literature and discussing their implications for threat assessment, the results will be examined in terms of the four hypotheses around which the study was structured.

*(1). The associations between making threats and approach will differ when the underlying motivation is one of grievance, compared with cases where the motivation is the seeking of a relationship.*

In the aggrieved, those making indirect threats were significantly more likely to approach, whereas the reverse was the case in the relationship seekers (refer to Table 3). A similar differentiation was found in terms of the association between number of indirect threats and approach (Table 4). The aggrieved showed significant associations with approach in overall number of threats, number of direct threats, number of conditional threats, and number of categories of threat used. There were no such associations in the relationship seekers. The “scheming” threat category was significantly associated with approach in the aggrieved, but not in the relationship seekers (refer to Table 5). Threats of physical violence were associated with approach in the aggrieved. There was a trend for the reverse in the relationship seekers and, although this difference did not reach statistical significance in this sample, the effect size was −0.286 (refer to Table 6). The use of abusive language in the threats was associated with approach in the aggrieved: in the relationship seekers, the converse did not reach statistical significance, and the effect size was –0.232. Approach in the aggrieved was associated with the clarity of threatened consequence, but this did not apply in the relationship seekers (refer to Table 7). In addition, in cases where threats had been made, approach in the aggrieved was associated with the length of intrusive behaviour, the number of modes of communication used, and with the presence of major mental disorder. These associations were absent in relationship seekers (refer to Table 7). In summary, important differences were found between the aggrieved and relationship seekers in terms of associations between threats and approach.

*(2). Associations identified between threats and approach in individual motivational groups will no longer be evident, if all the different motivational groups are considered together as one single group.*

The results provided evidence that consideration of the general sample without accounting for motivation is likely to disguise significant differences which exist – in other words, that the study of sets of cases based on a sector of employment, such as politicians or celebrities, which combine groups with different threat profiles, flatten out the results and disguise real differences. The number of indirect threats made was significantly associated with approach in the aggrieved, but with non-approach in the relationship seekers: yet, no such differences appear in the whole mixed motivations sample (Table 4). Schemers are significantly more likely to approach in the aggrieved, but not in the relationship seekers, yet, no difference is found in the mixed motivation sample (Table 5). Threats of physical violence are significantly associated with approach in the aggrieved and there is a trend to non-approach in the relationship seekers: yet, no such significant differences are found when all motivations are taken together (Table 6). The use of abusive language is significantly associated with approach in the aggrieved, but not in relationship seekers: no association is present in the mixed motivation group. The same applies to the threats being clearly defined (Table 7). The length of intrusive behaviour is strongly associated with approach in the aggrieved group, and in the mixed motivation sample, but not in the relationship seekers. The same pattern applies to the number of modes of communication. In those that made threats, approach is significantly associated with the presence of major mental disorder, but not in relationship seekers or the mixed motivations sample.

*(3). Between motivational groups, different associations with approach will be found according to the immediate purpose of making threats.*

Sufficient numbers for analysis were present for two of the Mullen threat classification categories (Table 5). A significant difference was found, in that scheming was significantly associated with approach in the aggrieved, but not in the relationship seekers. In both the aggrieved and the relationship seekers, shocking was associated with non-approach. This will be discussed further later.

*(4). The form of harm threatened may be linked with the likelihood of subsequent approach.*

It is notable that threats of physical violence were significantly associated with approach in the aggrieved, with a trend towards non-approach in the relationship seekers (refer to Table 6). However, there was little indication of any differences with threats of persistence, escalation, legal action, or suicide. No significant differences were found for three further items, threats of reputational damage, cases where the threats all containing the same consequences, or the number of different consequences threatened. However, the effect sizes in these items indicate that the relationships are worthy of further exploration in larger samples.

In summary, the results offered considerable support to the hypotheses under consideration. Our contention would be that consideration of the risk of adverse outcomes in those who make threatening or otherwise concerning intrusions into the lives of those in the public eye would benefit from incorporating the principals elucidated for the evaluation of risk in stalking, harassment, and threats in other populations (Mullen et al., 1999; MacKenzie et al., 2009). In other words, the top level in the hierarchy of examination should be the consideration of underlying motivation, rather than the nature of the victims’ employment. Furthermore, assessment should recognise that risk factors will differ according to the particular form of potential adverse outcome concerned. These principals apply equally well to the study of the making of threats as they do to other forms of harassing communication and physical intrusion. With threats, there is a third major element to consider and that is the question as to the immediate purpose of the threat to the threatener. Issues of mode and form of threat require consideration, but are generally subsidiary to the above.

The issue concerning motivation, to put it at its most banal, is that those motivated by love are unlikely to behave in the same way as those motivated by hate. In this study, it has been shown that risk factors associated with moving from threat to approach differ in important ways between those motivated by seeking a relationship and those fuelled by grievance. Lumping all motivations into one, as in other studies described above, blurs differences, as might be expected by the proverbial mixing of apples and oranges. A few characteristics are held in common, but many are not. The difference in conclusions between previous studies of celebrities and those of politicians can be explained by the predominance of a different motivation – relationship-seeking with celebrity cases and grievance with politicians. Differences in findings between studies within one sector (such as in threats and approach in celebrity cases between Dietz et al., 1991a and Meloy et al., 2008) are likely to be accounted for by variations in the motivational mix of the samples, different definitions of risk, and the difficulties of the retrospective study of case files assembled for other purposes.

The study is unusual, in that it used a classification for the immediate purpose of the threats made. Of particular interest, here is the finding that the Mullen classificational group, “shockers,” is firmly associated with non-approach in both the motivational groups studied: indeed, in this comparatively small sample, only 5% of “shockers” of any motivation went on to approach. “Shockers” are the cases where the threats were made to inflict emotional pain. This would appear to be an end in itself, which is not associated with a need for further action. This finding is linked to the differentiation made by Calhoun (1998) between “hunters” and “howlers,” in other words, those who “shout” and those who go on to act, which is also reflective of the differentiation between making and posing a threat. The finding about “shockers” may also be key to understanding some of the differences between the findings of this motivational study and those of samples of politicians, where the making of threats was negatively associated with the approach (Dietz et al., 1991b; Scalora et al., 2002a,b). In those studies, the most frequent motivation would appear to be that of grievance, although the samples are a mix of motivations. Our suggestion is that the cases included sufficient a proportion of “shockers” as to determine a significant association between threats and non-approach. Some evidence for this can be found in the details of the studies. For instance, in the study of members of congress by Dietz et al. (1991b), in which threats were significantly associated with non-approach, there were significant negative associations between approach and “attempts to instil fear in the politician,” “attempts to frighten the politician,” “attempts to instil worry in the politician,” and “attempts to provoke upset in the politician.” These items would appear to be encompassed by the Mullen “shocker” category. In the studies by Dietz et al. of inappropriate communications to celebrities, these data points do not appear. Yet, it is notable that only 5% of respondents cast themselves in the role of “enemies,” compared with 40% in the study of members of congress. No relation between threat and approach was found in this study. It may be that the relative absence of “shockers” in this sample determined the difference in this respect between the politicians and celebrities. It is also possible that the inclusion of ‘shockers’ in samples may have affected the findings in terms of a lack of association between direct threats and approach: for, direct threats are likely to be over-represented amongst ‘shockers’, given that indirect or veiled threats have less in the way of shock quality.

Other general observations from the study are that threats are very common in the study sample, being present in 77.8% of cases – and significantly more common in the aggrieved than the relationship seekers. In the aggrieved, but not in the relationship-seeking, length of intrusive behaviour and number of modes of communication were significantly associated with approach, as was mental disorder. The first two of these might be taken as an indication of persistence, rather than intensity, as it is of note that expressions of immediacy (e.g., deadlines) and indications of recent escalation were not associated with approach in those who made threats. Nor did the indicators of plausibility or implausibility show any significant effects. It might be theorised that issues of commitment, immediacy, and practicality are subsumed to some degrees within the question as to the immediate purpose of a threat: or that plausibility is of less import to an individual who is mentally ill. Finally, a significant association between mental disorder and approach in those that threatened was found in the aggrieved, but not in the relationship seekers, although the proportion of cases with mental disorder was greater in the latter. A possible explanation is that the nature of the mental illness may differ between the two motivational groups, with a greater proportion of delusional disorders in the aggrieved, and of schizophrenia in the relationship seekers. The findings of the study in terms of threats and the associations of approach are summarised in Table 8 for the ease of comparison of the “profiles” of the aggrieved and relationship seekers.

**TABLE 8:** Summary of differences in significant associations with approach between aggrieved and relationship-seekers threateners.

Aggrieved	Relationship seekers
**Significant associations with approach**	
***Types and numbers of threats***	
Indirect threats made (*p* 0.024, φ 0.316)	*No* indirect threats made (*p* 0.033, φ −0.329)
More threats made (*p* 0.036, φ 0.300)	NS* (φ −0.059)
More direct threats made (*p* 0.018, φ 0.332)	NS (φ −0.078)
More indirect threats made (*p* 0.008, φ 0.369)	*Fewer* indirect threats made (*p* 0.039, φ −0.318)
More conditional threats made (*p* 0.018, φ 0.329)	NS (φ 0.060)
More threat types used (*p* 0.025, φ 0.313)	NS (φ −0.115)
***Mullen threat categories***	
Schemers (*p* 0.024, φ 0.316)	NS (φ 0.145)
Not shockers (*p* 0.041, φ –0.301)	Not shockers (*p* 0.009, φ −0.426)
***Consequences threatened***	
Threats of physical violence (*p* 0.024, φ 0.316)	NS (Trend towards *no* threats of physical violence: φ –0.286)
***Language used in threats***	
Threats delivered in spoken form (*p* 0.043, φ 0.315)	NS (φ 0.232)
Use of conditional conjunctions (*p* 0.024, φ 0.316)	NS (Trend towards use of conditional conjunctions: φ 0.230)
Use of abusive language in threats (*p* 0.006, φ 0.388)	NS (φ −0.199)
Clearly defined threatened action (*p* 0.006, φ 0.383)	NS (φ −0.081)
***Mental disorder***	
Presence of major mental disorder (*p* 0.019, φ 0.351)	NS (φ −0.033)
***Persistence***	
Duration of intrusive behaviour (*p* 0.007, φ 0.401)	NS (Trend towards duration of intrusive behaviour: φ 0.304)
Number of different modes of communication used (*p* 0.002, φ 0.468)	NS (φ 0.136)

*NS = no significant difference.

## Limitations

The limitations to this study are self-evident. The sample size, at 126 cases, was relatively small, particularly in the sub-groups, and significant associations are likely to have been missed which would have become apparent, had a larger sample been used. In addition, there were insufficient numbers to examine all the categories in the motivational typology, or all those in the typology of intent. Some of these limitations may have been offset to a degree by the use of effect sizes as additional indices, and by the improved methodological purity and exactitude when compared with previous studies. It would evidently be desirable to repeat the exercise with larger numbers and in samples of different origin, despite the complexities and time that would be involved. Sampling procedures in future studies could usefully be designed in method to ensure roughly equal numbers of threateners and non-threateners. Second, this study did not examine violence directly, but rather approach as a proxy for violence. The use of a proxy was unavoidable given the low base rate of violence, and the choice of approach as a proxy at least enabled comparison with previous studies.

## Conclusion

As to what this study can contribute to the consideration of grievance-fuelled violence, (the expanding literature on which this is not the place to summarise), it provides a few small pieces to a very much larger jigsaw. It is through the availability of such comparative material, from different fields, that improvement may be made in the identification of risk factors to incorporate into population-based preventative approaches. The latter remain relatively primitive in that they aim to apply a rather coarse filter to an understanding of motives and interactions which are affected and modified by highly individual factors and events in a person’s life which cannot readily be captured through forms of screening: and the low base rate of violence makes the proportion of false positives high in any such screening process. Nevertheless, screening based on risk factors remains the principal tool available, and further consideration of individual and dynamic risk factors that cannot be incorporated into the design of such screens can later be applied to cases “screened-in” and to the design of interventions at an individual level.

The study dealt with the specific issues of the making of threats and their relevance to risk of approach, as a proxy for violence. Perhaps, its main contribution is methodological. Our principal conclusion would be that differences in motivation and in immediate purpose of threats should both be incorporated into future studies in the area of threats to people in the public eye, and other groups besides. We have suggested ways in which this could be done.

## Data availability statement

The datasets presented in this article are available to suitably qualified academic researchers upon request to the lead author.

## Author contributions

DJ designed the project, co-ordinated the analyses, and was principal writer. FF, PA, AM, CS, and AW contributed equally to the various stages of the project. All authors contributed to the article and approved the submitted version.

## Footnotes

1
www.fixatedthreat.com
